# Primary Yolk Sac Tumor of the Stomach in a 2-Year-Old Boy: Case Report on a Rare Site for Extragonadal Malignant Germ Cell Tumor

**DOI:** 10.1155/crpe/1148505

**Published:** 2025-08-20

**Authors:** Saiesh V. Reddy, Subhash Yadav, Samreen S. Qureshi, Sajid S. Qureshi

**Affiliations:** ^1^Division of Paediatric Surgical Oncology, Department of Surgical Oncology, Tata Memorial Hospital and Advanced Centre for Training Research and Education in Cancer (ACTREC), Tata Memorial Centre, Mumbai, India; ^2^Department of Pathology, Tata Memorial Hospital and Advanced Centre for Training Research and Education in Cancer (ACTREC), Tata Memorial Centre, Mumbai, India; ^3^Medical and Health Sciences, Homi Bhabha National Institute (HBNI), Mumbai, India

**Keywords:** alpha-fetoprotein, case report, germ cell tumors, pediatric, stomach, yolk sac tumor

## Abstract

Up to one-third of germ cell tumors are extragonadal neoplasms, with yolk sac tumors (YSTs) being the most common malignant histology. This report describes the successful multimodal management of a primary YST of the stomach in a 2-year-old boy. The child presented with melena, and further evaluation revealed a mass lesion in the cardia of the stomach. A biopsy established a diagnosis of a YST, which correlated with an elevated serum alpha-fetoprotein level. The child received three cycles of chemotherapy consisting of cisplatin, etoposide, and bleomycin (PEB), followed by surgical resection in the form of proximal gastrectomy. After an uneventful recovery from surgery, he received an additional three cycles of PEB and has remained disease-free for 7 years. This case highlights the potential for the occurrence of a relatively rare childhood tumor at an unusual site, which can pose diagnostic challenges. However, careful evaluation and meticulous management can lead to favorable outcomes.

## 1. Introduction

Pediatric germ cell tumors (GCTs) account for approximately 1%–3% of malignancies in this age group [1]. These tumors have distinct characteristics in terms of their locations and presentations. In children, there is a higher prevalence of extragonadal disease, which often affects areas such as the sacrococcygeal region, mediastinum, retroperitoneum, and other para-axial locations [2]. Less than 1% of GCTs occur in organs such as the liver, kidney, vagina, and stomach [1, 2]. Among the extragonadal sites, yolk sac tumor (YST) is the most frequently observed malignant histology. These tumors often show elevated levels of alpha-fetoprotein (AFP), which serves as a marker for persistent or recurrent disease [3]. YSTs exhibit characteristic histological features, with four general patterns and several recognized variations [2]. The pseudopapillary (or festoon) pattern and the microcystic (or reticular) pattern are among the most common. They contain Schiller–Duval bodies, which are structures comprised of a small central blood vessel surrounded by two layers of tumor cells. In terms of immunohistochemistry (IHC), YSTs typically stain positively for AFP, Glypican-3, and SALL4 [2].

While teratomas are frequently found in the stomach, with over 100 cases reported in infants and children, pure primary gastric YST is rare [4]. To our knowledge, only three cases of pure YST in children have been reported [5–7]. This report presents a case of a primary gastric YST in a 2-year-old child who was managed with surgery and chemotherapy at a tertiary cancer care center.

## 2. Case Report

**Figure 1:**
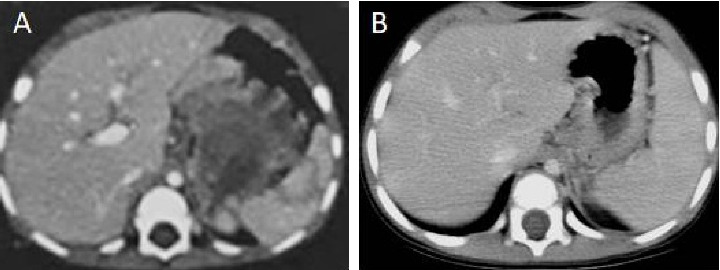
A computed tomography scan three cycles before chemotherapy reveals a large, heterogeneous mass in the gastrohepatic region (A). Significant reduction in lesion size was observed after chemotherapy, with disease now involving the proximal body of the stomach (B).

**Figure 2:**
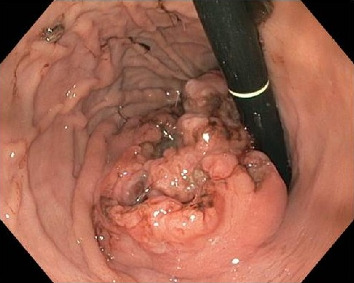
Esophagogastroduodenoscopy showed an infiltrative lesion with central excavation involving the fundus and proximal body of the stomach (retroflexed view).

**Figure 3:**
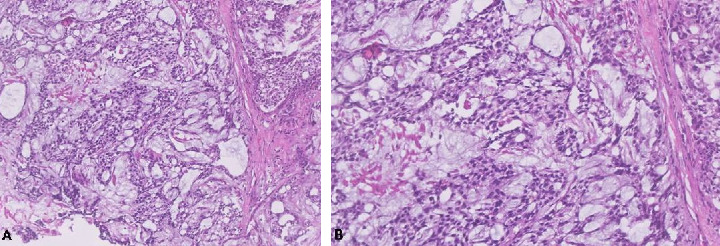
A biopsy taken from the stomach at diagnosis (hematoxylin and eosin staining, at 10x magnification) revealed a tumor organized in microcystic and glandular patterns within a myxoid and focally hyalinized stroma (A). Additionally, the tumor exhibited moderate nuclear atypia and eosinophilic cytoplasm (hematoxylin and eosin staining, at 20x magnification) (B).

A 2-year-old male child, born at full term, presented with a history of passing tarry stools for the past 2 months. Prior evaluation at a local center revealed a significant drop in hemoglobin (5 gm/dL), necessitating transfusions of three packed cell units. A thorough physical examination was unremarkable. A computed tomography (CT) scan identified a heterogeneous mass lesion in the gastrohepatic region, with possible origins from either the stomach or the pancreas (Figure 1(A)). No metastases were seen in the lungs. An esophagogastroduodenoscopy revealed a submucosal infiltrating lesion affecting the fundus and proximal body of the stomach, extending to the esophagogastric junction and exhibiting central excavation (Figure 2). A biopsy of the lesion showed features characteristic of YST (Figure 3).

Additionally, an elevation in serum AFP was detected at 191,912.5 ng/mL, while a scrotal ultrasound examination of the testes showed normal findings. These findings indicated stage III extragonadal GCT. The child then received three cycles of chemotherapy followed by surgical evaluation and three additional cycles of adjuvant therapy. In the first cycle, the child received etoposide (500 mg/m^2^ per cycle) and cisplatin (100 mg/m^2^ per cycle), followed by the addition of bleomycin (15 mg/m^2^ per cycle) in the subsequent two cycles (PEB), without significant adverse effects. By this time, the AFP had decreased to 467 ng/mL, corresponding with a substantial mass reduction on a response assessment CT scan (Figure 1(B)). The child then underwent a proximal gastrectomy via a left thoracoabdominal approach. During the surgery, the stomach was divided using a linear cutter stapling device, while the esophagus was clamped with a Satinsky clamp. A continuous hand-sewn esophagogastric anastomosis was created with a 4-0 polydioxanone suture. Postoperatively, the child's recovery was uneventful, and he was discharged on postoperative day 10.

**Figure 4:**
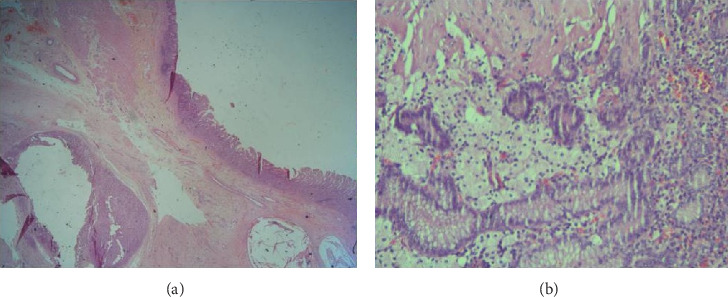
Histopathological examination of the resected specimen (hematoxylin and eosin, magnification 10x) reveals residual tumor in the submucosa of the stomach (a), along with Schiller–Duval bodies (hematoxylin and eosin, magnification 20x) (b).

Histopathological examination of the specimen revealed scant residual GCT, consisting of 90% YST and a 10% mature teratomatous component (Figure 4). The tumor had infiltrated the submucosa and muscularis propria with extensive chemotherapy-induced changes, clear margins, and reactive lymph nodes (0/16). A repeat AFP assay showed a further reduction postsurgery to 31 ng/mL. The child completed the remainder of the chemotherapy (three cycles of PEB), which led to the normalization of AFP levels (2 ng/mL). The patient remains asymptomatic and disease-free, clinically and biochemically, at a 7-year follow-up.

## 3. Discussion

Extragonadal GCTs typically occur in midline sites in the following order of frequency: sacrococcygeal, mediastinal (including pericardium, heart, and lung), intracranial, retroperitoneal, and uterine regions [1, 2]. Primary gastric GCTs are exceedingly rare, and most of the reported cases were teratomas [4]. A comprehensive literature review uncovered three case reports from India of primary pure gastric YSTs in children (Table 1). One case involved a 1.5-year-old child who had elevated serum AFP levels that aided in the diagnosis. Another case reported a 3-year-old boy with a lesion in the lesser curvature of the stomach, along with omental deposits and liver metastases. This child was treated with PEB chemotherapy and underwent sleeve gastrectomy. The final case described a 2.5-year-old boy with a lesion in the proximal stomach, perilesional deposits, and mild ascites [5–7].

The rarity of malignancies in the upper gastrointestinal tract among children leads to a diagnostic challenge. The uncertainty in distinguishing the origin of the tumor on radiology, whether from the stomach or the pancreas, compounds this confusion. However, characteristic histopathological findings from a biopsy, along with elevated AFP levels, confirmed the diagnosis of a GCT. AFP serves as a critical tumor marker, being highly sensitive for both diagnosis and monitoring during and after treatment for GCTs. A failure to normalize or an increase in AFP levels may suggest incomplete tumor resection or recurrence, even before imaging shows any abnormalities [3]. Surgery is the primary treatment for GCTs. Following our institutional protocol for extragonadal tumors, the patient underwent a total of six cycles of PEB chemotherapy, three cycles before surgery and three cycles afterward. Although long-term follow-up data on previously reported children with gastric GCTs is limited, our patient has remained disease-free without experiencing treatment-related side effects for 7 years.

## 4. Conclusion

Extragonadal GCTs arising from atypical sites such as the stomach can present with diagnostic and therapeutic challenges. Careful clinical judgment, along with a systematic approach to investigations including imaging, tumor marker assays, and tissue diagnosis, is crucial in achieving an accurate diagnosis and developing an effective treatment plan.

## Figures and Tables

**Table 1:** Literature overview.

Authors/year	Age/gender	Stomach site	AFP levels	Stage	Treatment	Status	Follow-up
Baruah et al. [5], 2007	1.5/male	Proximal stomach	Elevated	NA	NA	NA	NA
Mandelia et al. [6], 2018	3/male	Lesser curvature	21,000 ng/mL	4	Chemotherapy + surgery	Alive	3 months
Guru et al. [7], 2019	2.5/male	Proximal stomach	19,100 IU/mL	3	Chemotherapy + surgery	Alive	NA
This report, 2025	2.5/male	Proximal stomach	191,912.5 ng/mL	3	Chemotherapy + surgery	Alive	7 years

## Data Availability

The data that support the findings of this study are available on request from the corresponding author. The data are not publicly available due to privacy or ethical restrictions.
